# Surface display of Salmonella epitopes in *Escherichia coli *and *Staphylococcus carnosus*

**DOI:** 10.1186/1475-2859-10-22

**Published:** 2011-04-11

**Authors:** Nguyen Thanh Nhan, Ernesto Gonzalez de Valdivia, Martin Gustavsson, Truong Nam Hai, Gen Larsson

**Affiliations:** 1 Division of Bioprocess Technology, School of Biotechnology, Royal Institute of Technology (KTH), SE-106 91 Stockholm, Sweden; 2 Vietnam Institute of Biotechnology (IBT), Vietnamese Academy of Science and Technology (VAST), Hanoi, Vietnam

## Abstract

**Background:**

*Salmonella enterica *serotype Enteritidis (SE) is considered to be one of the most potent pathogenic *Salmonella *serotypes causing food-borne disease in humans. Since a live bacterial vaccine based on surface display of antigens has many advantages over traditional vaccines, we have studied the surface display of the SE antigenic proteins, H:gm and SefA in *Escherichia coli *by the β-autotransporter system, AIDA. This procedure was compared to protein translocation in *Staphylococcus carnosus*, using a staphylococci hybrid vector earlier developed for surface display of other vaccine epitopes.

**Results:**

Both SefA and H:gm were translocated to the outer membrane in *Escherichia coli*. SefA was expressed to full length but H:gm was shorter than expected, probably due to a proteolytic cleavage of the N-terminal during passage either through the periplasm or over the membrane. FACS analysis confirmed that SefA was facing the extracellular environment, but this could not be conclusively established for H:gm since the N-terminal detection tag (His_6_) was cleaved off. Polyclonal salmonella antibodies confirmed the sustained antibody-antigen binding towards both proteins. The surface expression data from *Staphylococcus carnosus *suggested that the H:gm and SefA proteins were transported to the cell wall since the detection marker was displayed by FACS analysis.

**Conclusion:**

Apart from the accumulated knowledge and the existence of a wealth of equipment and techniques, the results indicate the selection of *E. coli *for further studies for surface expression of salmonella antigens. Surface expression of the full length protein facing the cell environment was positively proven by standard analysis, and the FACS signal comparison to expression in *Staphylococcus carnosus *shows that the distribution of the surface protein on each cell was comparatively very narrow in *E. coli*, the *E. coli *outer membrane molecules can serve as an adjuvant for the surface antigenic proteins and multimeric forms of the SefA protein were detected which would probably be positive for the realisation of a strong antigenic property. The detection of specific and similar proteolytic cleavage patterns for both the proteins provides a further starting point for the investigation and development of the *Escherichia coli *AIDA autotransporter efficiency.

## Background

Bacterial display of proteins on the cell surface using recombinant DNA technology has been used in microbiology, biotechnology and vaccine technology and has been an area of intense research since reports of this novel technology were published [1-5]. One of the most studied areas of application has been live vaccine delivery through the surface display of antigenic proteins [2,4]. This technique gives potential advantages over traditional vaccines since they are less costly to produce (there is no need for extensive isolation and purification) [2], they are better recognised by the host immune system and therefore create better immune responses [3], they elicit strong long-lasting immunity [2], components of the host, e.g. outer membrane lipopolysaccharides (specifically in *E. coli*), may contribute to a very strong immune response acting as an adjuvant to the recombinant antigenic proteins [3], and finally the surface expression may also be safer than attenuated or inactivated vaccines because bacteria strains used for surface expression must be non-pathogenic.

Several surface display systems for both gram negative and gram positive bacteria have been described in the literature but the detailed knowledge of these systems varies considerably [5]. Many naturally occurring proteins have been developed as carriers for a target protein, such as outer membrane proteins, lipoproteins, secreted proteins and subunits of surface appendages. These systems show some disadvantages, for example with respect to the size of foreign proteins or high sensitivity to the structure of inserts [6,7]. *E. coli *has attracted a lot of interest due to its easy handling and the wealth of literature and knowledge accumulated. The successful transplantation of the pathogenic *E. coli *β-autotransporter (type V secretors of Enteropathogenic bacteria) to laboratory strains has provided a new technique for vaccine production through exposure of antigens on the surface [6,8,9]. This transport vehicle is the most abundantly expressed protein transporter for surface display in these cells and the "AIDA" autotransporter (Adhesin Involved in Diffuse Adherence) [10] was chosen for the present work. The original vector contains all the parts necessary for translocation to the environment and consists of its own unique elements: an AIDA-specific signal sequence, a passenger protein, a linker region known to influence translocation, and a translocation unit (AIDA^C^) intended for insertion as a system-specific pore in the outer membrane (C-terminal). A suggested transport mechanism for the AIDA autotransporter was described and the fate of the passenger is to stay anchored or to be cleaved-off [6]. It is generally believed that the passenger protein must be kept in an unfolded state to achieve proper translocation across the outer membrane [6].

*Staphylococcus carnosus* is a Gram-positive bacterium frequently used for protein display and it is considered to be a food-grade bacterium due to its low DNA homology with the pathogenic strain, *Staphylococcus aureus*. The *S. carnosus *surface display vector uses the M and X domains of protein A from *Staphylococcus aureus *(SpA) [11] where the latter contains a charged repetitive domain responsible for binding to cell-wall peptidoglycan.

In this work we wished to explore the possibilities of expression of salmonella proteins using the *E. coli *AIDA autotransporter, in order to understand its potential for surface exposure and to compare this to surface expression in the Gram positive bacterium *Staphylococcus carnosus*, earlier reported to be used in live-vaccine applications [5]. We believed this to be particularly interesting since *E. coli *is structurally similar to salmonella and the production strain is a K12 strain, which lacks the O-antigen. We selected the H:gm flagellar protein, recognized and targeted by both the innate and adaptive immune systems [12,13], and the SefA fimbrial protein, that is restricted to SE and other closely related group D Salmonella, due to their central role in salmonella infections [14]. We show that both proteins can be translocated by both *E. coli *and *S. carnosus *but with differing efficiencies and characteristics.

## Materials and methods

### Bacterial strains and plasmids

**Figure 1:**
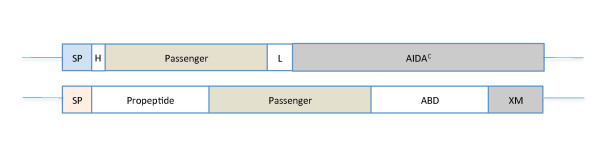
**Schematic representation of the vectors used in this work**. Above: structure of the *E. coli *vector with the anchoring domain AIDA^C^, a linker region (L), a His_6 _tag (H) and the specific signal sequence for the AIDA system (SP). Below: structure of the staphylococcal surface display vector with the anchoring regions (X, M), the albumin-binding domain (ABD), a propeptide and the signal sequence from *S. hyicus *(SP). The passenger is either SefA or H:gm in either vector. The N-terminal is facing to the left and the anchoring C-terminal to the right. The relative sizes of the vector parts are approximate.

*Escherichia coli *strain O:17 ΔOmpT [15] and *Staphylococcus carnosus *strain TM300 [16] were used as host cells for the surface display of foreign peptides. The *E. coli *strain was deleted with respect to OmpT, a part of a strategy to remove the cleavage of the target protein from the surface. The *E. coli *surface display vector, pDT1, is a derivative of pBR322 under the control of the constitutive AIDA promoter and the AIDA-specific signal sequence. The proteins are expressed as fusions to a N-terminal positioned His_6 _tag [9]. The staphylococcal surface display vector, pSCZ1, was derived from the original plasmid pSCX*m *as earlier described [11]. The vector contains, apart from the cell-wall-anchoring XM regions from *S. aureus*, an albumin-binding domain used for detection purposes. The signal sequence is derived from *Staphylococcus hyicus*. The principal outline of both vectors is shown in Figure 1.

Recombinant plasmids were constructed using standard recombinant DNA techniques [17]. Two derivatives of pET32 containing the genes for gm and sefA, respectively, were used as templates for amplification of the gm and sefA genes. Amplification was done using DreamTaq DNA polymerase (Fermentas) using standard reaction conditions for 30 cycles. The primers that were used for amplification are listed in table 1. Plasmids for both expression systems were isolated (QIAprep^® ^Spin Miniprep Kit, Qiagen) and then cleaved by appropriate restriction enzymes (Fermentas). PCR DNA and a Gel band Purification Kit (GE Healthcare, UK) were used for purification of DNA from amplification or enzymatic reactions. The two different vectors were ligated at room temperature for two hours and then transformed to *E. coli *and *S. carnosus*, respectively. Transformants were selected on Luria-Bertani medium agar plates using the appropriate antibiotics. The recombinant DNA sequences, obtained by subcloning, were thereafter sequenced (Eurofins MWG Biotech AG, Germany). The sefA insert was found to contain one silent mutation (456G>T) compared to the sequence X98516 published on GenBank. For gm, two nucleotide substitutions (506G>C, 763A>G) were found compared to the GenBank sequence AY353533, resulting in two amino acid substitutions (G169V and K255E). Since these substitutions were present in both the *E. coli *and *S. carnosus *vectors they are likely also present in the pET32-gm template. An additional substitution (23A>T) giving rise to an amino acid change (N8I) was present in the *E. coli *vector, likely due to random error in the amplification. Oligonucleotide primers were synthesized by Eurofins MWG Synthesis GmbH (Germany).

**Table 1:** Primers used for amplification of gm and sefA

Oligo name	Sequence
pDT1_gm_forw	AAA CAC GTG GGG CAC AAG TCA TTA ATA CAA ACA G
pDT1_gm_rev	AAA TCT AGA GCA CGC AGT AAA GAG AGG ACG
pDT1_sefA_forw	AAA CAC GTG GGG CTG GCT TTG TTG GTA ACA AAG
pDT1_sefA_rev	AGG TCT AGA GCG TTT TGA TAC TGC TGA ACG TAG
pSCZ1_gm_forw	GGG CTC GAG GCA CAA GTC ATT AAT ACA AAC AG
pSCZ1_gm_rev	AAA GTC GAC ACG CAG TAA AGA GAG GAC G
pSCZ1_sefA_forw	ATA CTC GAG GCT GGC TTT GTT GGT AAC AAA G
pSCZ1_sefA_rev	ATA GTC GAC GTT TTG ATA CTG CTG AAC GTA G

### Cultivation

A 2.5 μl volume of *E. coli *cells was taken from the -80°C storage and inoculated into 25 ml LB medium (Scharlau Chemie S. A.) with 100 μg/ml ampicillin. The cells were cultivated overnight in 250 ml baffled shake flasks at 37°C and stirred at 180 rpm. Recombinant cells of *S. carnosus *with either of the two proteins were taken from glycerol stocks at -80°C and used to inoculate 25 ml of LB medium with chloramphenicol to 10 μg/ml. The culture was grown overnight at 37°C and 150 rpm in a 250 ml baffled shake flask. OD_600_ was used to monitor the growth (Novaspec II).

### Isolation of outer membrane protein

Outer membrane proteins from *E. coli *strains were extracted according to the literature [18]. The outer membrane proteins were stored at -20°C before analysis.

### Gel electrophoresis (SDS-PAGE 10%)

Samples mixed with reducing buffer (0.0625 M Tris-HCl, pH 6.8, 20 g/l sodium dodecyl sulfate (SDS), 43% glycerol, 10% (v/v) 2-mercaptoethanol, 10 mg/ml bromophenol blue) were heated at 95°C for 10 minutes and analyzed on NuPAGE^® ^10% Bis Tris Gels (Invitrogen) in MOPS buffer [18]. Gels obtained after SDS-PAGE were stained with Coomassie Brilliant Blue solution (7% acetic acid, 50% methanol and 2.5 g/l Coomasie Brilliant Blue R-250). SeeBlue^® ^Plus2 Prestained Standard (Invitrogen) was used to assess the molecular weights of the protein.

### Western blot for AIDA^C ^detection

Western blot detection was based on antibodies to the AIDA^C ^domain fused to either of the target proteins. The SDS-PAGE gel, the nitrocellulose membrane and filter papers were first equilibrated in transblotting buffer (2.93 g l^-1 ^glycine, 5.81 g l^-1 ^Tris-HCl, 0.37 g l^-1 ^SDS, 20% methanol), blotted at 15 V for 45 min (Trans-blot^® ^SD Semidry transfer cell machine, Bio-Rad) and blocked in phosphate buffered saline, PBS (9 g l^-1 ^NaCl, 0.21 g l^-1 ^KH_2_PO_4_, 0.726 g l^-1 ^Na_2_HPO_4_, pH 7.4) and 5% low fat milk for about one hour. The membrane was washed three times with PBS buffer (10 min each) before being incubated at room temperature for one hour with rabbit serum IgG specific for the AIDA^C^, diluted 1:50000 in PBS with 0.1% bovine serum albumin (BSA). After being rinsed three times with PBS to remove non-specific binding (10 min each), the membrane was incubated for one hour with alkaline phosphatase-linked goat anti-rabbit IgG secondary antibody (Sigma) diluted 1:10 000 in PBS. The membrane was then again washed three times with PBS. The substrate (FAST^™ ^BCIP/NBT, 5-bromo-4-chloro-3-indolyl phosphate/nitro blue tetrazolium, Sigma) was dissolved in 10 ml of deionized H_2_O and poured onto the membrane to detect the antigen-antibody complex through alkaline phosphatase cleavage of the substrate, resulting in a dark brown staining of the membrane.

### Western blot for detection of the His_6 _tag

The proteins derived from *E. coli *were fused to an N-terminal His_6 _tag which can be used for detection by commercial antibodies. The procedure was the same as that described for the detection of AIDA^C ^but with the use of antibodies and detection by the HisProbe-HRP and SuperSignal system (Pierce). Blots were scanned by a Fujifilm Image Reader 1000 V1.2 (Fujifilm Life Science) using the software: Image gauge 4.0.

### Labeling for FACS analysis

The procedure for flow cytometry analysis has been described before [19]. The proteins were visualised by fluorescence markers either to the *E. coli *His_6 _tag or to the albumin-binding domain (ABD) in *S. carnosus*. A sample of 10 μl of an overnight culture was incubated with biotinylated 6× His tag-specific rabbit polyclonal antibody (Abcam) and a streptavidin-AlexaFluor^488 ^conjugate (Invitrogen). The labelled cells were resuspended in ice-cold PBS and protected from light until FACS analysis was performed (FACS Vantage SE, BD Biosciences). The procedure was repeated in a similar manner for *S. carnosus *except that the human serum albumin binding to the protein was evaluated with HSA-Alexa^647 ^(HSA conjugate solution, Invitrogen).

## Results and discussion

### Expression of H:gm and SefA in *E. coli*

**Figure 2:**
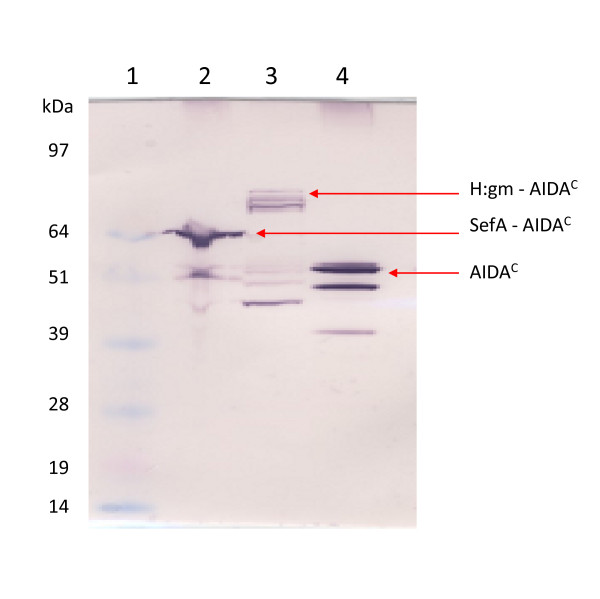
**Expression of the salmonella epitopes in *E. coli***. Western blot showing fusion proteins to the AIDA^C ^translocator. Lane 1: Marker, Lane 2: SefA, Lane 3: H:gm, and Lane 4: Negative control (AIDA^C ^without fusion partner). Arrows indicate the calculated sizes. AIDA^C^-specific antibodies were used for detection.

*E. coli *O:17 was cultivated in a batch process with cells expressing the target proteins with the AIDA autotransport system. Samples of the cell extract were taken for Western blot analysis using AIDA^C ^antibodies for target protein detection. The individual proteins were His_6 _tagged in the N-terminal and fused to the AIDA^C ^translocator in the C-terminal (Figure 1) and the resulting molecular weights of the fusion proteins were; H:gm (104 kDa), SefA (64 kDa) and AIDA^C ^(51 kDa). From the blots shown in Figure 2, it can be seen that SefA was located at approximately 64 kDa and was thus produced at the correct size. The H:gm-AIDA^C^, on the other hand, was detected with a molecular weight somewhat lower than expected. Protein fragments of the fusion protein, binding AIDA^C^, were also detected in this preparation. In the empty vector containing only AIDA^C ^and no passenger the protein was detected at full length, but also here smaller proteins with AIDA^C ^affinity were seen. These data show that the passenger proteins were produced as fusions to the translocation unit of AIDA^C ^although they were also partly degraded, particularly in the case of the H:gm production. We have used an OmpT background which we have previously shown will reduce the cleavage from the surface and also of the AIDA^C ^unit. Since a lower molecular weight than that of AIDA^C ^was detected, it is evident that the mutant cannot altogether prevent this degradation.

### Distribution of protein in the cellular fractions in *E. coli*

**Figure 3:**
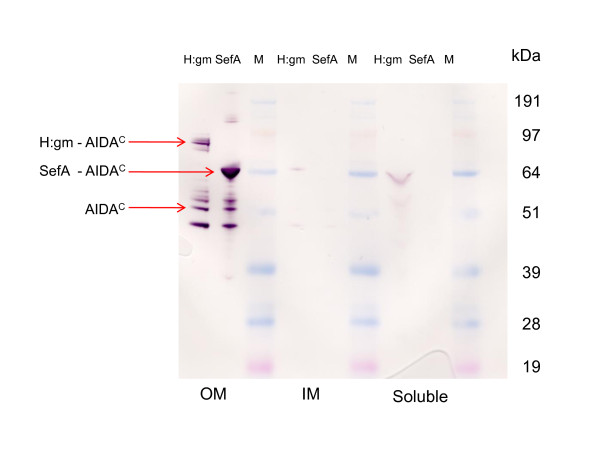
**Distribution of the target proteins in cellular compartments of *E. coli***. Western blot of cell disintegrate was separated into outer membrane (OM), inner membrane (IM) and soluble fractions. Western blots of the separate samples as detected by the AIDA^C ^specific antibody interaction.

In order to understand whether or not the target proteins were fully translocated to the outer membrane (OM), cellular fractions were isolated with an emphasis on the outer membrane fraction. Figure 3 shows the results. It is noted that H:gm was exclusively found in the outer membrane fraction while SefA seemed to be present in small amounts in both the soluble and inner membrane fractions (IM). These fragments are even smaller in size than those translocated to the OM. Their presence might be due to insufficient isolation of the OM membrane protein fraction or to cytoplasmic cleavage of the N-terminal signal sequence part of the protein. In either case, this part is negligible and we draw the conclusion that almost all the protein had been successfully transferred to the OM. The outer membrane fraction contains, however, a defined number of different fusions to the AIDA^C ^translocator, i.e. a clear proteolytic pattern can be distinguished for both proteins. This pattern seems strikingly similar for both proteins and it is suggested that the cleavage is partly carried out by the same proteases for both the proteins. The degradation takes place either in the periplasm or in the OM since an uncleaved N-terminal signal must have been present for periplasmic and OM transport. Proteolysis can however often occur during cell break-up and sample preparation. We have used a standard commercial protease cocktail which improved the situation (data not shown) but we could not completely prevent the proteolysis. One could argue that the larger size of the H:gm protein compared to SefA, 53 and 14,4 kDa respectively, and the fact that the N-terminal is the last to leave the cytoplasm [6,10], makes periplasmic degradation by e.g. DegP more probable but, since the pattern of proteolysis is similar for both proteins, the size difference, and consequently the longer time appearance in the periplasm, does not seem to be a likely explanation. OM cleavage seems to be a more likely mechanism. In the Western blot of SefA, two other bands were also detected in the OM fraction at molecular weights of 130 kDa and 200 kDa. Literature data on SefA expression indicate that this fimbrial structural protein usually tends to aggregate to form dimers or trimers, which could well agree with the protein sizes on the blot. We have confirmed that these forms also occur when the protein is expressed in periplasm (unpublished data). It is realized that these oligomer forms could be particularly interesting for the induction of a very strong immune response.

### FACS analysis of *E. coli*

Although OM expression was confirmed, the H:gm and SefA proteins might be subjected to two possible steric orientations: directed either inwards towards the periplasm or outwards towards the extracellular environment. The latter is the preferred, although the generation of efficient antibodies to evoke an immunological response cannot be conclusively established without further specifically designed experiments.

**Figure 4:**
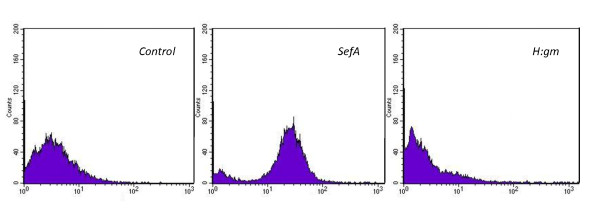
**Flow cytometry analysis of *E. coli *expression of salmonella antigens**. *E. coli *O:17 expression of the fusion proteins. The negative control consists of cells carrying the empty plasmid. Fusion proteins were detected by a biotinylated His tag-specific antibody conjugated to Streptavidin-Alexa^488^.

Flow cytometry with binding to the reporter systems was used to confirm the orientation of the fusion proteins. Since the plasmid is designed with a His_6_-tag, it will consequently be expressed in the N-terminal of the protein on the cell surface in an orientation mostly outwards towards the environment. The His_6 _tag antibody binding was detected by the fluorescence of streptavidin-Alexa^488^. Data are presented in the histograms of Figure 4 and, compared with the control, there is a distinct signal showing that the SefA protein is oriented outwards in contrast to the H:gm. The signal confirms a very narrow distribution of the fluorescence intensity from each cell, indicating that each cell expresses more or less the same amount of protein.

**Figure 5:**
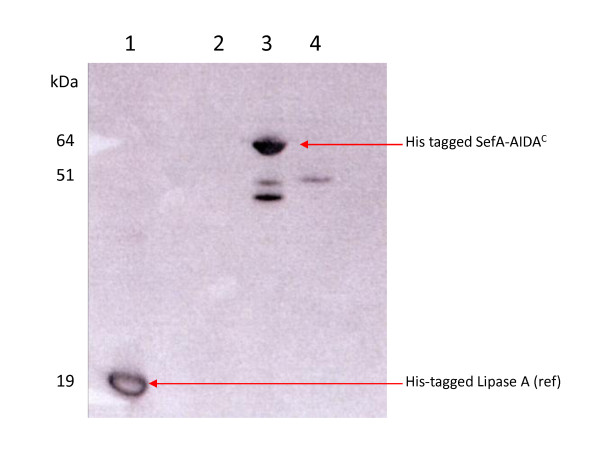
**Presence of the His_6 _tag in fusion proteins located in the outer membrane fraction of *E. coli***. Western blot showing: Lane 1: Positive control; Bacillus Lipase 19.3 kDa [37]; Lane 2: H:gm; Lane 3: SefA; and Lane 4: Negative control: AIDA^C^. All proteins carry the His_6 _tag. The molecular sizes of marker proteins are indicated to the left (kDa). Western blot detection with Abcam 6× His tag specific antibody and HisProbe™-HRP.

However, since we know that both proteins are present in the OM but that the size of the H:gm fusion protein is lower than expected, we suggest that proteolytic cleavage of the N-terminus, removing the His tag, has taken place. This would mean that conjugation of the antibodies to this tag could not take place and there would consequently be no signal in any of the His-tag-based analytical systems. We therefore performed a new Western blot using antibodies against the His_6 _tag. As shown in Figure 5, the His-tag is present only in connection with the SefA protein and not in the H:gm protein fraction. The actual orientation of the truncated H:gm protein cannot however be clarified with these data since the truncated forms may also still be facing the environment.

**Figure 6:**
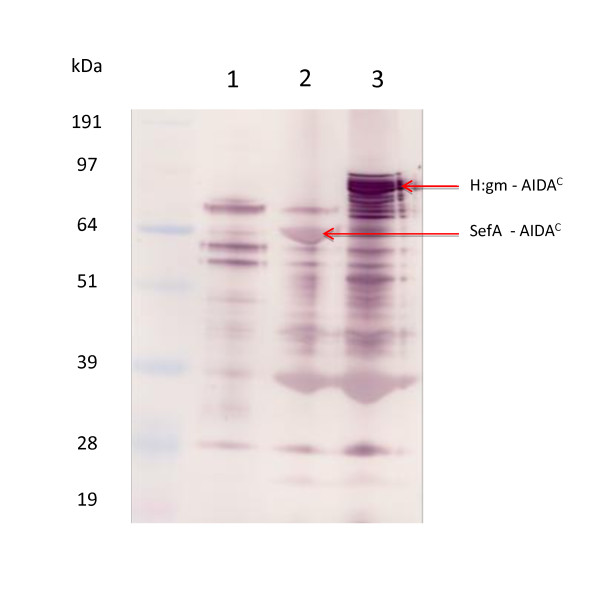
**Polyclonal salmonella antibody binding to *E. coli *outer membrane fraction expressing the salmonella epitopes SefA and H:gm**. Western blot data showing in Lane 1: Control/empty vector, Lane 2: SefA expression, and Lane 3: H:gm expression.

The SefA protein is obviously a possible candidate for live vaccine generation, but the question as to whether or not H:gm can be used can only be answered after further analysis of the orientation. The lack of a small portion of the fusion protein does not *per se *disqualify the construct for the desired purpose if the remainder is still facing the extracellular environment. That the gm protein displayed contains two amino acid substitutions in the *S. carnosus *vector and three in the *E. coli *vector must be kept in mind, however. To better understand the protein quality in this respect, we investigated its binding to polyclonal antibodies raised towards *Salmonella enteritides *(SE). The Western blot is shown in Figure 6. The results shows that several of the *E. coli *outer membrane proteins are recognised by the SE antibodies, which is not surprising given the close relationship between salmonella and *E. coli*. The binding includes the proteins of sizes earlier detected by antibodies towards the AIDA^C ^translocator (Figure 3), i.e. there is a matching of the sizes determined as SefA and H:gm epitopes.

### FACS analysis of *Staphylococcus carnosus*

To compare the *E. coli *expression levels with a more common expression system used for vaccine production, *S. carnosus *surface expression was used for the same two salmonella proteins, H:gm and SefA. *S. carnosus *has earlier attracted considerable interest due to the possibility of using a one-step simple translocation, and the solidity of the cell wall allows robust production and handling. The thick cell wall of Gram-positive bacteria has however been a potential drawback leading to low frequency of transformation. Recently, however, electroporation-mediated transformation in *S. carnosus *has been optimised, and this potentially circumvents this problem. The anchoring to the cell wall is here provided by the X and M domains from *Staphylococcus aureus *protein A (SpA), where X is a charged repetitive domain binding to the cell wall and M consists of an LPXTG motif followed by hydrophobic amino acids and a short charged tail.

**Figure 7:**
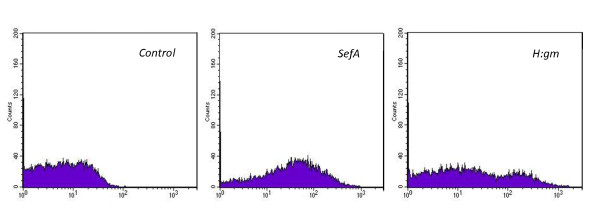
**Flow cytometry analysis of *S. carnosus *expression of salmonella antigens**.* S. carnosus *TM300 expression of fusion proteins. The negative control carries an empty plasmid. Fusion proteins were detected by binding to human serum albumin HSA conjugated to Streptavidin-Alexa^647^.

The reporter proteins were detected by flow cytometry as shown in Figure 7, where both the staphylococcal clones gave positive peaks not present in the control. The detection is here based on the binding of a fluorophore towards the albumin-binding domain positioned closest to the anchoring regions in the cell wall. The actual presence of the recombinant H:gm and SefA proteins cannot thus be certified. The peak observed in the histogram for H:gm was very broad, which may even indicate the presence of two populations, of which the major part is not expressing the protein. In any case, the wide spectrum indicates a large expression variation amongst the cells in the population. In this system also, the H:gm protein seems to be more difficult to express.

## Conclusion

Two genes representing proteins of one salmonella fimbrium and one flagellum were inserted to full length and within the correct open reading frame within both *E. coli *and *S. carnosus *vectors. Proteins were expressed and readily transferred to the outer membrane and cell wall, respectively. One of the proteins, SefA, was conclusively shown to face the external environment to its full length in *E. coli*, but in the case of the other protein, H:gm, a degradation form was produced and the localisation could not be fully ascertained. Polyclonal antibodies to *Salmonella enteritides *bound to both protein antigens and, despite the degradation and unproven localisation of H:gm, the experiment showed that they were recognised by the antibody. The possibility that the *E. coli *expressed proteins to generate antibodies from a host immune response has not however been shown for either system, and it will have to be studied in further experiments. The further binding of the salmonella antibodies to several proteins in *E. coli *could imply that this could serve as an adjuvant for recombinant antigenic proteins and this would thus promote the use of this host cell. The expression in *E. coli *also seems to be the most promising due to the very narrow distribution of protein on each cell (SefA). In addition, the probable existence of multimeric forms of the SefA protein in *E. coli *could promote the development of a strong and potent antigenic property. Further, we believe that to overcome the partial proteolytic degradation in *E. coli *is a more straightforward research and development route than the formulation of a hypothesis as to why *S. carnosus *expression is so very broad. The similarity between the proteolytic patterns shown from both SefA and H:gm protein expression in *E. coli *will form an interesting basis for further research and optimisation of the system.

## Competing interests

The authors declare that they have no competing interests.

## Authors' contributions

NTTN performed the major part of the experimental work and contributed to the manuscript; MG contributed to the experimental work and the supervision of the work; EGdeV supervised the cloning and contributed to the manuscript; TNHAI and GL were responsible for the original concept and GL supervised the experiments and wrote the manuscript. All authors have read and approved the manuscript.
